# The Cognitive Transaction: Toward a Human Factors Research Agenda for AI in Anesthesia and Perioperative Care

**DOI:** 10.2196/102683

**Published:** 2026-09-10

**Authors:** Sheena Warner, Christopher H Stucky, Tamara Haegerich, Young J Yauger

**Affiliations:** 1 Nell Hodgson Woodruff School of Nursing, Emory University, 1520 Clifton Road NE, Atlanta, GA, 30322, United States, 1 (404) 727-7980; 2 Brian D Allgood Army Community Hospital, Pyeongtaek, Republic of Korea; 3 American Association of Nurse Anesthesiology Foundation, Rosemont, IL, United States

**Keywords:** cognitive transaction, perioperative artificial intelligence, human–artificial intelligence interaction, human-AI interaction, human factors, clinical decision support, anesthesia informatics

## Abstract

AI is now embedded in the infrastructure of perioperative care. Risk stratification algorithms, hemodynamic prediction tools, and clinical decision support systems are active in operating rooms at major health systems, and their adoption is accelerating. However, the field has studied model performance and organizational implementation while largely bypassing the moment between them: the real-time encounter in which an anesthesia provider must decide, under active case conditions, what to do with an AI-generated output. We term this the cognitive transaction and argue that it is the fundamental unit of perioperative AI implementation. The perioperative environment presents a specific constellation of conditions that existing human-AI interaction research was not designed to address. Continuous real-time decision demands, extreme time compression, high cognitive load, and consequences that unfold in seconds distinguish the operating room from the clinical contexts where most provider-AI interaction research has been conducted. What we know about AI adoption in radiology, oncology, or ambulatory care does not readily translate to this setting. The cognitive moment in anesthesia has its own structure, its own failure modes, and its own research requirements. This paper examines what those requirements are. We analyze how the operating room functions as a pre-existing human-machine cognitive system into which AI is now being inserted, and why the conditions of that system generate predictable vulnerabilities: miscalibrated trust, automation bias, and cognitive friction produced by interfaces optimized for technical accuracy rather than clinical usability. We argue that these failure modes are not incidental but structural and that they will persist regardless of model performance until the provider-AI interaction is itself treated as a research object. We identify 4 priority research domains. The first concerns the structure of provider-AI disagreement and the methods needed to distinguish automation bias from legitimate clinical insight. The second concerns the longitudinal dynamics of trust calibration across repeated clinical encounters rather than single-session experimental designs. The third concerns interface design for high-acuity workflows, specifically what constitutes a usable AI output for a provider managing a patient in real time. The fourth concerns the need for ecologically valid study designs capable of capturing provider reasoning under actual perioperative conditions rather than retrospective or survey-based proxies. The anesthesia and perioperative research community is positioned to lead this work. The clinical specificity, domain knowledge, and professional stake required to design meaningful studies are all present within the field. Evaluating the cognitive transaction under perioperative conditions, not the computational model in isolation, is both a methodological imperative and a patient safety priority.

## Introduction

This paper articulates a human factors research agenda addressing 3 levels of clinical AI integration. The primary target domain is the perioperative surgical environment, a uniquely complex setting characterized by continuous real-time decision demands, extreme time compression, and high cognitive load [1]. Furthermore, unlike insular environments such as an aviation cockpit, the operating room functions as a highly connected socio-spatial network [2]. Introducing AI into this intricate spatial and social architecture requires a dedicated human factors approach to ensure that new technologies align with actual clinical workflows. The insights derived from this environment function less as a direct template for clinical AI generally than as a stress test: if human-AI collaboration can be made to work under the extreme time compression and cognitive load of an active operating room case, the resulting principles are more likely to hold with even greater confidence in less demanding clinical AI contexts. Finally, this work offers a theoretical contribution by positioning the “cognitive transaction” (the real-time encounter between algorithmic output and clinical judgment) as the fundamental unit of analysis for safe and effective AI deployment.

## The Gap Nobody Is Naming

Perioperative health care is generating more data, more rapidly, than any individual clinician can process. The tools being built to close that gap are arriving faster than the science needed to deploy them safely. AI has entered the perioperative environment not as a future possibility but as present infrastructure. Risk stratification algorithms, early warning systems, and clinical decision support modules are increasingly embedded in the electronic health record platforms that anesthesia providers navigate daily.

The research literature evaluating these tools has expanded accordingly, with a growing body of studies reporting model performance and prospective validation results. A parallel implementation science literature has grown alongside it, examining organizational adoption, clinician behavior change, and system-level barriers to uptake. What neither body of literature has systematically studied is the moment between the two.

An anesthesia provider, mid-case, encounters an AI-generated risk output, such as a predicted probability of hemodynamic instability or a hemorrhage risk score embedded in the obstetric record. The number appears, and the provider must decide in seconds, under high cognitive load and with competing attentional demands, what to do with it. Do they act on it, override it, or adjust their existing assessment? The model has done its work; now the human must do theirs.

That cognitive transaction, the encounter between algorithmic output and clinical judgment in real time, is the fundamental unit of perioperative AI implementation. Remarkably, it is also the unit that the field has studied most inconsistently.

This omission does not stem from a lack of interest. Researchers across clinical AI, human factors engineering, and implementation science have recognized that provider-AI interaction shapes outcomes in ways that model performance alone cannot predict. However, the perioperative environment presents a specific constellation of conditions that makes it categorically different from the clinical contexts where most human-AI interaction research has been conducted. These unique conditions include continuous real-time decision demands, extreme physiologic complexity, and consequences that unfold in seconds rather than hours. The cognitive moment in anesthesia has its own structure, its own failure modes, and its own research requirements. Until those requirements are met, the gap between model performance and clinical value will persist regardless of how good the models are.

## Why Perioperative AI Is a Distinctive Case

The perioperative environment requires its own analytical framework, not an adaptation of existing clinical AI research conducted elsewhere. Anesthesia providers do not encounter technology as an occasional resource; rather, they operate within it continuously. A single case requires the simultaneous management of a ventilator, infusion pumps, a physiologic monitoring array, and an anesthesia information management system. Each of these components generates real-time data streams, requires ongoing interpretation, and demands an immediate response when conditions deviate from clinical expectations. This is not a new condition introduced by AI. It is the baseline cognitive architecture of the role.

Cook and Woods [3], writing on adaptation to new technology in the operating room, described this environment as one in which human and machine contributions are already interwoven in ways that cannot be disentangled without fundamentally altering the nature of the work. This baseline matters for AI implementation because it means the operating room is not a neutral surface onto which a decision support tool is introduced. It is an existing human-machine cognitive system, already structured by relationships of trust, workload distribution, and situational awareness, into which an AI layer is being inserted [3,4].

The question is not whether providers can tolerate an additional data source. They tolerate many. The question is what happens to the existing cognitive architecture when one of those data sources is probabilistic, opaque, and carries an implicit authority that physiologic monitors do not.

To an anesthesia provider, a pulse oximeter reading of 88% is self-evident. An AI-generated risk probability of 0.73 is not. A pulse oximeter reading speaks directly to a physiologic state that the provider can verify and act upon. An AI risk probability speaks to a statistical relationship between patient characteristics and an outcome, derived from a training cohort the provider did not choose, using features the provider may not be able to inspect, with a CI that is rarely displayed. Every anesthesia provider knows how to respond to one. Almost none have been trained to critically evaluate the other. Treating these 2 outputs as equivalent in design, training, or implementation is a category error with clinical consequences.

The Human-AI Joint Cognitive Systems (HAIJCS) framework, proposed by Xu and Gao [5] and applied to the obstetric anesthesia context by Warner et al [6], offers a conceptual foundation for understanding this distinction. Under HAIJCS, the human provider and the AI system are understood as collaborative cognitive agents, each contributing to shared situational awareness, task execution, and decision-making under the authority of the human. The relevant question is not only whether the model predicts correctly but also whether the human-AI dyad operating together under actual clinical conditions reaches better decisions than either would reach alone and whether the conditions of the operating room allow that dyad to function as designed.

The Joint Cognitive System (JCS) and its HAIJCS extension model the operating room at the level of the system: a standing architecture of shared situational awareness, trust, and control between human and machine agents [4-6]. The cognitive transaction operates at a different grain. It is not a second name for that system-level relationship; it is the discrete event the relationship depends on, and none of the frameworks it draws on treat that event as an object of study in its own right. Trust calibration, as JCS frames it, describes a general disposition the human holds toward the machine over time. Situation awareness, as modeled by Schulz et al [4] in anesthesia, describes an ongoing process of perceiving, comprehending, and projecting system states. The cognitive transaction is neither the disposition nor the process. It is the specific encounter, this output, this patient, this second, in which trust gets tested and situational awareness gets updated. Distributed cognition makes a related claim at the structural level: cognitive work is located across people, tools, and environment rather than inside any single agent [7]. All 3 describe standing conditions of the joint system. None isolates the moment of integration as a unit that can be observed and measured directly. The cognitive transaction is that unit.

Research from aviation and military operations has established that high-workload, time-pressured environments generate predictable patterns in human-automation interaction, including automation bias, complacency, and degraded monitoring of system states during periods of high task demand [8,9]. Perioperative AI introduces an epistemic challenge that those findings were not built to address. The provider must act on a prediction they cannot verify in real time, about a future state that may never materialize, for a patient who may bear little resemblance to the population from which the model learned. Instruments can be independently cross-checked against observable measurements; risk scores cannot be directly verified in the same way. Translating human factors insights from adjacent domains is a necessary starting point, not a sufficient end point. Perioperative AI implementation requires a research agenda built for the operating room, not borrowed from the cockpit.

## What the Current Literature Misses

The clinical AI literature has made substantial progress on model development, producing tools that perform well on retrospective datasets. Reviews of perioperative AI consistently demonstrate improvements in predictive accuracy for outcomes including postoperative complications, intraoperative hypotension, and adverse events across multiple patient populations [10]. This is meaningful work, but model development solves only a fraction of the problem.

A striking pattern has emerged from the broader clinical AI literature: AI systems working independently have begun to outperform clinicians using those same systems as decision support tools [11]. Explanations for this range from automation neglect to a fundamental mismatch between how models communicate uncertainty and how clinicians process it in practice. While the authors acknowledge that this evidence is early and drawn largely from controlled settings, it reveals a critical reality. Giving clinicians access to an AI tool does not automatically produce better decisions, and some have argued that the assistive model itself may be fundamentally flawed as a design paradigm [12]. Regardless, the underlying observation stands. The quality of the human-AI interaction, rather than the model’s isolated performance, determines whether the combination is synergistic, additive, or subtractive. This interaction has rarely been studied with the rigor applied to the models themselves.

The implementation science literature has done important work naming this problem. Studies on sepsis decision support tools have found alert fatigue, high override rates, and clinician abandonment of models that appeared predictive on paper [13,14]. These deployment failures are not primarily technical; they are cognitive and interpretive. Clinicians cannot inspect the key features driving a prediction or assess if the training cohort matches the patient in front of them. When a model’s output conflicts with clinical assessment, clinicians may lack a clear framework for determining which source of information to trust. The result is either uncritical acceptance, which introduces automation bias, or reflexive dismissal, which undermines the tool’s purpose [15].

Reviews have summarized technology and human factors that influence clinician-AI interaction, such as data quality, performance, transparency, expertise, cognitive biases, and trust [16]. Perioperative AI researchers have identified similar concerns [10,17]; however, the specific perioperative environment makes failures involving technology and human factors particularly acute. Three features of the perioperative environment explain why that methodological work is both especially difficult and necessary.

First, deliberation time is not merely compressed but functionally eliminated during critical perioperative events. Other clinical AI contexts deploy decision support in real time, including sepsis alerts, opioid risk prediction, and chronic disease management, but providers in those settings retain a meaningful window for reflection, consultation, and staged response [13,14,18]. A perioperative risk output during an evolving hemodynamic event does not allow for reflection, consultation, or staged response. Cognitive psychology research illustrates that time pressure can reduce the quality of activities, modify risk-taking, change underlying cognitive processing, and increase the relative relevance of different information sources [19]; hence, understanding how time pressure influences the clinician-AI interaction in the context of anesthesia and operative events is critically important. Second, human-AI interaction research has rarely accounted for the profound cognitive load of active case management, where fatigue, divided attention, and the dynamic spatial environment of the surgical team dictate whether an alert registers as a signal or mere noise [4,17]. Third, anesthesia providers lack established norms for interacting with probabilistic AI algorithms. Unlike radiologists, who have worked alongside AI-assisted image review tools for years, anesthesia providers are encountering these tools without a shared professional vocabulary for evaluating, trusting, or overriding them [20,21].

This third feature points to a structural explanation for the literature gap. The clinician-researchers best positioned to study the cognitive moment in perioperative AI, those who understand both the perioperative environment and the methodological tools required to study human-AI interaction, exist at an intersection that the field has not yet organized around. Computational researchers lack deep access to perioperative decision-making processes. Implementation scientists lack the anesthesia-specific domain knowledge to recognize what makes perioperative AI distinctive. Meanwhile, the anesthesia providers who navigate these cognitive demands daily have not traditionally received training in human factors or informatics. The gap is not simply a gap in knowledge. It is a gap in who is asking the question.

## Toward a Research Agenda for the Cognitive Moment

Rather than proposing a comprehensive framework, this agenda outlines priority research questions grounded in the distinctive features of the perioperative environment. These domains are drawn primarily from risk-stratification and predictive-alert use cases, which currently represent the AI applications most deeply embedded in perioperative workflows. Real-time hemodynamic prediction tools compress the transaction further, leaving less room for the reflective divergence discussed below. Generative systems, which produce free-text recommendations rather than a single score, introduce additional interpretive demands that the disagreement and interface-design frameworks discussed here do not yet address. Whether the cognitive transaction holds the same structure across these AI types, or requires type-specific variants of each research question, is an open question this agenda raises rather than resolves. The most pressing concern is the structure of provider-AI disagreement: when an anesthesia provider and an AI system arrive at different risk assessments for the same patient, what explains the divergence? Current methodologies, largely adapted from radiology and emergency medicine, focus primarily on agreement metrics rather than investigating these underlying sources of conflict [22]. In the operating room, provider divergence from an AI output may reflect automation bias, but it may equally signal critical clinical insight inaccessible to the model, a patient’s affect, a subtle physiological pattern recognized over thousands of cases, or unrecorded institutional variables. A perioperative research agenda must disentangle these sources of divergence rather than collapsing them into a binary agreement metric. Capturing this nuance requires mixed methods designs that pair quantitative performance data with structured qualitative inquiry into provider reasoning, an approach largely absent from the current literature. The critical decision method developed by Klein et al [23] offers an established route to this distinction: a structured retrospective interview technique that reconstructs the cues, prior cases, and contextual signals a provider drew on at the moment of divergence, rather than treating the divergence itself as the end point of analysis. It remains one of the most commonly applied cognitive task analysis techniques in clinical decision-making research, spanning surgical and critical care settings [24].

Trust in an AI system is fluid and not a fixed attribute. However, the current clinical AI literature predominantly evaluates trust through cross-sectional surveys or single-encounter experimental designs, yielding a static snapshot. This fails to capture the most vital question for perioperative implementation: How does trust in a specific AI system develop, erode, and recalibrate over repeated clinical encounters? Anesthesia providers who override a model’s output and later find it correct will reasonably become skeptical of the model’s future reliability. On the other hand, providers who defer to the model and observe a good outcome may default to uncritical reliance. Longitudinal designs that pair momentary, in-the-moment reliance judgments with case-level outcome tracking, sampled repeatedly across a provider’s caseload, would let calibration be measured as a trajectory rather than a single point estimate. The relevant construct is not trust in the abstract but calibrated reliance: whether a provider’s confidence in a given output corresponds to its actual reliability on a case-by-case basis.

The perioperative AI literature has given little systematic attention to how model outputs should be communicated to providers operating under the cognitive conditions of active case management. Current tools tend to present a risk score or classification optimized for technical legibility rather than clinical usability, prioritizing mathematical accuracy over the ability of a multitasking clinician to quickly and appropriately integrate the information. Human factors research has long established that the design of information presentation shapes clinical behavior just as much as the information itself. Outputs developed without regard for cognitive load, task context, and provider mental models routinely fail at the point of care, even when the underlying algorithm performs well [4,13]. What constitutes a usable, low-friction AI interface for an anesthesia provider mid-case, in terms of format, timing, level of specificity, and fit with existing workflow, remains unanswered. One example of a low-friction output might surface as a color-coded flag integrated directly into the existing vital sign display rather than a separate probability score requiring independent interpretation. Another might be a brief natural language prompt, such as “rising hemorrhage risk, consider early type and screen,” delivered at a natural pause in the case rather than immediately when the model updates. Adjudicating between formats requires usability testing under simulated time pressure, not static comprehension surveys. Solving this for the extreme constraints of the operating room will establish baseline principles for AI usability across all clinical environments. Until this is addressed, interface design will continue to be driven by technical conventions rather than clinical evidence.

Ultimately, the field requires research methodologies capable of capturing not only what anesthesia providers think about AI in the abstract but also how they reason about and integrate these outputs within ecologically valid clinical contexts. This should encompass both perceptual and deliberative processes. Expert anesthesia providers often detect physiological deterioration through pattern recognition that precedes conscious reasoning. Recent work pairing physical simulation with eye tracking has begun to capture this in critical care, finding that clinicians’ visual attention did not reliably increase even when an AI recommendation was unsafe [25]. Perioperative research needs the same instrumentation applied to the compressed conditions of an active case. This necessitates moving beyond retrospective analyses and survey-based designs. Instead, research should use realistic clinical simulations incorporating model outputs and evolving patient data to examine the continuous cognitive process of integration, rather than focusing solely on the final binary decision. We must investigate how providers respond to unexpected algorithmic predictors, how the framing of an output shapes clinical action, and precisely when and why human judgment diverges from a model’s recommendation. These are not peripheral questions of user experience. They are the empirical foundation on which safe, deployable perioperative AI must be built.

## Why This Matters Now

Perioperative AI is not a future abstraction; it is a present reality. Tools for perioperative risk prediction, hemodynamic management, and postoperative complication forecasting are already embedded in clinical workflows at major health systems, and their adoption is accelerating [10]. The window for shaping how these systems are designed, evaluated, and deployed is open now, and it will not remain open indefinitely. Once these tools become embedded in institutional infrastructure, clinical workflows reorganize around them, and the cost of redesign increases substantially. The decisions being made today about what perioperative AI looks like, what it communicates, and how it interacts with provider judgment will permanently shape the cognitive environment of anesthesia practice.

The research agenda proposed here is not a precondition for building these systems; it is a precondition for building them safely and effectively. Models can be designed and deployed without understanding how providers interact with their outputs. In fact, they already have been. The consequence, documented repeatedly in adjacent clinical AI contexts, is a generation of tools that perform well in evaluation but fail in practice. This occurs not because the underlying science was wrong, but because the implementation ignored the human at the other end of the interface [11,13].

Anesthesia providers are not passive recipients of this technology. They are, by the nature of their role, the final cognitive layer between an algorithmic output and a clinical action. That position is not incidental. It is the precise point at which the entire value proposition of perioperative AI either materializes or collapses. A research program that takes this position seriously, that asks what the provider needs to know, what the interface needs to communicate, and what the conditions are under which human and algorithmic judgment combine to produce outcomes better than either alone, is not a niche methodological interest. It is the central scientific problem of perioperative AI implementation.

The anesthesia and perioperative research community is uniquely positioned to lead this work. The clinical specificity required to design meaningful studies, the domain knowledge required to interpret findings, and the professional stake in getting implementation right are all present within the field. Engaging the anesthesia and perioperative community in the development of AI solutions is consistent with calls for the engagement of other specialties, such as primary care, in research and implementation efforts that affect their patient populations. Such engagement helps ensure that AI applications are developed in ways that address the needs of frontline practitioners and ultimately improve patient outcomes [26]. That leadership, however, must be exercised in partnership. The research priorities identified here sit at the intersection of cognitive psychology, health informatics, implementation science, and clinical practice. No single discipline has the tools, the access, and the domain knowledge to address them alone. Anesthesia providers bring irreplaceable expertise in the perioperative environment, cognitive psychologists bring the methodological frameworks for studying human-AI interaction, informaticists bring the technical infrastructure for capturing and analyzing clinical data, and implementation scientists bring the frameworks for translating findings into practice. In practical terms, each stakeholder has a concrete starting point: anesthesia providers can partner with cognitive psychologists to design the simulation studies this agenda calls for; health system informaticists can instrument existing AI tools to capture override rates and response times rather than waiting for purpose-built research infrastructure; and clinical AI developers can treat these research questions as design requirements to address before deployment, rather than as validation exercises conducted afterward. Underlying all of these is a shared recognition that the cognitive transaction at the intersection of algorithmic output and clinical judgment is itself a discrete research object, distinct from the model, the organization, and the provider in isolation. Naming it as such is the first necessary step toward studying it rigorously and designing perioperative AI systems that are not only accurate but also genuinely useful in the operating room.

## Abbreviations

HAIJCS: Human-AI Joint Cognitive Systems
JCS: Joint Cognitive System
